# Identification of critical residues of O-antigen-modifying *O*-acetyltransferase B (OacB) of *Shigella flexneri*

**DOI:** 10.1186/s12860-022-00415-8

**Published:** 2022-03-24

**Authors:** Munazza I. Rajput, Naresh K. Verma

**Affiliations:** Division of Biomedical Science and Biochemistry, Research School of Biology, The Australian National University, Bldg.134, Linnaeus Way, Canberra, ACT 2601 Australia

**Keywords:** Membrane protein, O-antigen, O-acetyltransferase, Serotype conversion, *Shigella flexneri*

## Abstract

**Background:**

Shigellosis is an acute gastrointestinal disease caused primarily by the bacterium *Shigella flexneri*. Upon ingestion, *S. flexneri* initiates a serotype-specific immune response that targets the O-antigen of the pathogen’s lipopolysaccharide. O-antigen subunits are modified by the addition of chemical moieties, which give rise to new serotypes of *S. flexneri*. Nineteen different serotypes of *S. flexneri* have been recognized. A recently identified O-antigen-modifying enzyme, *O*-acetyltransferase B (OacB), which adds an acetyl residue at either position 3 or 4 of Rhamnose^III^ (3/4-O-acetylation) in serotypes 1a, 1b, 2a, 5a, 7a, Y, and 6 and position 6 of N- acetylglucosamine (6-O-acetylation) in serotypes 2a, 3a, Y and Yv of the O-antigen subunits. Critical residues in other proteins involved in O-antigen modifications such as glucosyltransferases (Gtrs) and acetyltransferase (Oac) of *S. flexneri* have been identified, whereas identification of important amino acids in OacB function is yet to be determined.

**Results:**

Hydrophobicity analysis showed that OacB is a transmembrane protein with 11 transmembrane segments, 12 loops, and periplasmic N- and cytoplasmic C- termini. Bioinformatics analyses revealed that OacB contains acetyltransferase-3 domain and several conserved residues. Using site-directed mutagenesis, selected amino acids were mutated to alanine to elucidate their role in the mechanism of action of OacB. Seven amino acids R47, H58, F98, W71, R116, R119, and S146 were found critical for the OacB function.

**Conclusion:**

In the absence of a three-dimensional structure of the serotype converting enzyme, *O*-acetyltransferase B (OacB), a clear role of important residues in the mechanism of action is precluded. Therefore, in this study, using site-directed mutagenesis, seven residues critical to the function of OacB were identified. The lack of agglutination of cell expressing mutant OacB in the presence of the antiserum indicated the functional role of the corresponding residues. Hence, this study provides significant information about key residues in OacB which might be involved in forming the catalytic sites of this O-antigen modifying enzyme of *S. flexneri*.

**Supplementary Information:**

The online version contains supplementary material available at 10.1186/s12860-022-00415-8.

## Introduction

*Shigella flexneri*, the predominant causative organism of bacillary dysentery in developing countries is accountable for 270 million shigellosis cases with 212,000 deaths annually [1]. Nineteen serotypes of *S. flexneri* have been recognized and each serotype differs from the other based on variations in lipopolysaccharide (LPS) structure. LPS plays an important role in bacterial virulence and the O-antigen is its most distal and variable domain followed by a core oligosaccharide and a lipid A. O-antigen is an immunodominant part of LPS and plays an important role during the infection process by evoking a serotype-specific humoral and adaptive immune response in the human host, and any modification to the O-antigen helps bacteria escape the human immune system.

All serotypes of *S. flexneri* except serotype 6 bear a common O-antigen backbone of repeating tetrasaccharide units consisting of N-acetylglucosamine and three rhamnoses residues (serotype Y). This parent structure is modified by the addition of glucosyl, acetyl, or phosphoethanolamine residue(s) to one or more sugars to give rise to new serotypes [2, 3]. The O-antigen modification by addition of O-acetyl or glucosyl residues is carried out by temperate phages. These phages integrate into the chromosomes of the host to form prophages that stably express serotype conversion genes [4]*.* Whereas phosphoethanolamine transferase (Opt)-mediated O-antigen modification is plasmid-borne [5]. Recently identified O-antigen-modifying enzyme, O-acetyltransferase B (OacB), adds an acetyl residue at either position 3 or 4 of Rhamnose III (3/4-O-acetylation) in serotypes 1a, 1b, 2a, 5a, 7a, Y, 6, and position 6 of N- acetylglucosamine (6-O-acetylation) in serotypes 2a, 3a, Y and Yv of the O-antigen subunits [6]. The *oacB* gene is carried by a temperate bacteriophage Sf101 which has recently been isolated from a 1c strain of *S. flexneri* [7]. OacB is an integral membrane protein and belongs to Pfam family Pf01757 which is recognized as acetyltransferase-3 (AT3) domain proteins [7, 8]. This enzyme has unique characteristics of acetylating substrate (Rhamnose III of O-antigen) in the absence of any other associated enzyme or domain. The mechanism behind the O-acetylation of O-antigen by OacB is yet unknown and the absence of X-ray crystallographic data on OacB warrants identification of important residues present in this enzyme. Thus, the investigation of key features of OacB is needed to understand the mechanism of serotype conversion by OacB.

Therefore, in this study, potentially indispensable residues in OacB were identified by performing multiple alignments of OacB with other acetyltransferases proteins present in different serotypes of *S. flexneri* and other related bacteria. Residues identified were then mutated to a neutral amino acid using site-directed mutagenesis (SDM). *S. flexneri* strains with mutated OacB protein demonstrated an inability to react with serotype-specific antiserum when tested in a slide agglutination assay. The observed effect of mutant forms of this protein indicates possible catalytic or structural roles played by individual amino acids. Functional roles observed in this study can serve as a good starting point to identify residues forming acyl donor interaction sites in OacB.

## Methods

### Bacterial strains, plasmids, and growth conditions

Bacterial strains used in this study are listed in Additional files 1 and 2**.** All the plasmids in this study (Additional file 3) were propagated in *E. coli* strain JM109. Luria-Bertani (LB) broth was used to grow *E. coli* and *S. flexneri* bacterial cultures at 37 °C (*E. coli*) or 30 °C (*S. flexneri*) at 180 rpm (rpm). LBA medium supplemented with 100 μg/ml ampicillin was used to plate the bacterial cultures.

### DNA methods

PCR amplification was carried out using Pfu Ultra II HS DNA polymerase (Stratagene). All the oligonucleotides mentioned in Additional files 4 and 5 were synthesized by Sigma-Aldrich. Plasmid vectors were derived from pBAD/*Myc*-His A (Invitrogen). Promega miniprep DNA kit was used to isolate the plasmids using the methods described by Sambrook et al.*,* [9]. Details of the plasmid constructs made in this study are described in Additional file 3.

Standard protocols adapted from Sambrook et al.*,* were employed for cloning, electroporation, and colony PCR [9]. Restriction endonuclease digestions and ligation reactions were performed according to the manufacturer’s instructions. Site-directed mutagenesis was performed as recommended by the Stratagene QuikChange Site-directed Mutagenesis with the *PfuUltra II* Fusion HS DNA Polymerase kit (Stratagene). XLI blue electrocompetent cells were used for the transformation of mutated plasmids. Sanger sequencing was carried out to confirm the presence of a correct mutation at Biomolecular Resource Facility, John Curtin School of Medical Research, Australian National University.

### Cloning of *oacB* gene into pBAD/*Myc*-his a

*oacB* gene was cloned into pBAD/*Myc*-HisA vector using conventional cloning protocols available in Sambrook et al.*,* [9]. Colony PCR using Pfu polymerase (Promega) was used to amplify *oacB* gene from the SFL2535 strain of *S. flexneri* using specific primers pair oacB pBADF and oacB pBADR (Additional file 4)**.** PCR product was purified and cloned into expression vector pBAD/*Myc*-His A at the *Xho*I and *EcoR*I sites to create pNV2111. The recombinant plasmid was transformed into freshly prepared JM109 electrocompetent cells. The transformants were selected on LB plates supplemented with ampicillin (100 μg/ml). The presence of the insert in pNV2111 was further confirmed by Sanger sequencing.

### Cloning of erythromycin resistance gene in pNV2111

The erythromycin gene (*erm*) was amplified using Pfu polymerase (Promega) from plasmid pNV2074 using primer pairs Em^R^-SphI-Fwd and Em^R^-SphI-Rev (Additional file 4). Plasmid pNV2111 was digested with *Sph*I (New England Biolabs), dephosphorylated using calf intestinal alkaline phosphatase (CIAP) from Fermentas, and then gel purified. The ligation of vector and insert was achieved using T4 DNA ligase obtained from New England Biolabs. The ligation mix was transformed into freshly prepared JM109 cells and clones were selected on LB plates containing erythromycin (250 μg/ml). A clone was sequenced using Sanger sequencing to confirm the presence of the erythromycin gene and named as pNV2132. Furthermore, pNV2132 was transformed into *S. flexneri* strain SFL1691 (negative for *oacB*) electrocompetent cells to create recombinant strain SFL2572 for carrying out the functionality assay described below.

### Site-directed mutagenesis

The Quick-change Site-directed mutagenesis protocol was used to perform mutagenesis (Stratagene). Primers used to create mutations are presented in Additional file 4. The generated constructs were treated with *Dpn*I and transformed into XLI blue super competent cells to repair the nicks. Mutant constructs were sequenced entirely by Sanger sequencing using suitable primers to ensure intended mutations were introduced without the introduction of any other extra mutations during PCR.

### Bioinformatics analysis

The protein sequence of O-acetyltransferase B (OacB) was obtained from (accession no. AII27840.1) [7]. NCBI-CDD and pBLAST (https://blast.ncbi.nlm.nih.gov/Blast) were used to identify any conserved domain(s) by comparing it with similar proteins from other organisms evolutionarily related to *S. flexneri.* Clustal Omega (EMBL-EBI) [10] with default parameters was then used to perform multiple alignments of OacB with other acetyltransferase proteins.

### Topology prediction

TOPCON (http://topcons.net/), was used to examine the amino acid sequence of OacB for the presence of hydrophobic regions. TOPCON uses five subset methods, OCTOPUS (http://octopus.cbr.su.se), Philius (http://www.yeastrc.org/philius), Polyphobius (http://phobius.cgb.ki.se/poly.html.), SCAMPI (http://topcons.cbr.su.se/) and SPOCTOPUS (http://octopus.cbr.su.se/) to predict the presence of transmembrane and non-transmembrane regions in a protein [11].

### Preparation of O-acetylation specific antisera

Crude antiserum raised in sheep immunized with serotype 1c strain, SFL1683 (positive for *oacB*) was collected and purified to render it specific to 3/4 O-acetylated Rha III epitope. All the nonspecific antibodies in the antiserum were absorbed by mixing antiserum with the heat killed SFL1691 cells (*oacB* negative 1c strain). Prepared antiserum was employed in slide agglutination assay, and agglutination observed with strain SFL1683 within 1 min was considered as specific to 3/4 O-acetylation. No agglutination observed with SFL1691 further confirmed the antiserum specificity.

### Functionality assay OacB using slide agglutination

The functionality of OacB in pNV2132 was tested by electroporating the plasmids into *S. flexneri* serotype 1c strain SFL1691 which was negative for *oacB* gene (as confirmed by its genome sequence). The transformants were selected on LB plates supplemented with erythromycin (250μg/ml) and incubated at 30 °C. Single colonies of SFL2572 were picked from the plates and added to a drop (10ul) of 3/4 O-acetyl-specific antiserum on a glass slide. Visible clumping of bacterial cells with the antiserum achieved within 1 min of swirling of the slide was considered as positive, which in turn indicated the presence of functional OacB. Levels of agglutination were compared against the clumping of positive control strain, SFL1683 (serotype 1c strain carrying *oacB* gene) within 1 min of the addition of antiserum. Agglutination recorded as high (+++), medium (++), low (+) or no agglutination (−). SFL1691 was used as negative control which showed no agglutination even after 1 min of the addition of antiserum. All the agglutination assays were performed in triplicates.

### OacB overexpression

Plasmid constructs were transformed into freshly prepared electrocompetent cells of TOP10 *E. coli*. Cultures were inoculated in LB containing ampicillin (100μg/mL) and incubated at 37 °C overnight with shaking at 180 rpm. Next day, 3 mL of the overnight starter cultures were added to 200 mL (1:66 dilution) of warm LB containing ampicillin (100 μg/ml) in a 500 mL Schott bottle and incubated at 37 °C at 180 rpm for approximately 3 h until optical density (OD_600_) reached 0.4. The cultures were then induced with 0.2% L-arabinose and grown for 4 h at 37 °C,180 rpm. The bacterial cultures were centrifuged at 10,000 x g for 15 min at 4 °C. After removing the supernatants, the cell pellets were washed (2X) with 10 mL of 30 mM Tris-HCL (pH 8.0) by centrifugation (3000 x g; 10 min; 4 °C). Pellets were suspended in 200 μl 20% sucrose (w/v) in 30 mM Tris-HCl (pH 8.1) and 20 μl of 1 mg/ml of lysozyme in 0.1 M EDTA (pH 7.3) and incubated on ice for 30 min. After incubation, 5 ml of ice-cold Milli Q water was added, and the cells were lysed by double-cycle in Emulsiflex-B15 (Avestin) high-pressure homogenizer. Cellular debris from all the samples was removed by centrifugation at 15,000 x g for 15 min at 4 °C. The supernatant free of cells was then centrifuged using 41 Ti rotors in an ultracentrifuge (Optima Xpn-100) at 100,000 x g for 60 min at 4 °C to collect membrane proteins. The supernatant was discarded, and the pellet was resuspended in 200 μl of Buffer A (50 mM Tris-HCl, (pH 8.0) containing 100 mM NaCl. The membrane protein samples were used immediately or stored at − 80 °C [12].

### Protein analysis

10 μl of 2 x sample buffer (0.5 M Tris-HCl, pH 6.8 containing 10% (w/v) SDS, 20% glycerol, 0.1% bromophenol blue) was added in a volume of prepared membrane protein samples containing 10 or 25 μg of protein. The amount of protein was quantified using the bicinchoninic acid protein (BCA) assay kit and the required volume was loaded onto BioRAD pre-cast SDS-PAGE gel (12–14%) for analysis.

### Coomassie staining

After SDS-PAGE gel electrophoresis of membrane protein samples, 200 ml Coomassie brilliant blue stain [0.1% (w/v)] was used to stain the gel overnight. The next day the gel was destained using 100 ml of destaining solution for 15–30 min.

### Western blot analysis

Directly after SDS-PAGE, the membrane protein samples were transferred to charged polyvinylidene fluoride (PVDF) membrane using the BioRad Trans-Blot Transfer Cell apparatus according to the manufacturer’s instructions. Once the transfer was complete, the membrane was soaked in 5% (w/v) skim milk (Diploma) prepared in phosphate buffer saline for blocking for 1 h or overnight. Following blocking, the membrane was washed 3 x for 15 min, in PBS containing 0.05% (v/v) Tween-20 (Sigma -Aldrich). After washing, the membrane was incubated with anti-His antibodies conjugated to horseradish peroxidase (R &D technologies) overnight.

The membrane was washed afterward three times (15 min each time) with PBS containing 0.05% (v/v) Tween-20. The reaction was observed under chemiluminescent camera dock (Vilber-Lourmat) and Vilber-Lourmat software version 15.11.

## Results

### Topology analysis of OacB using prediction programs

**Fig. 1:**
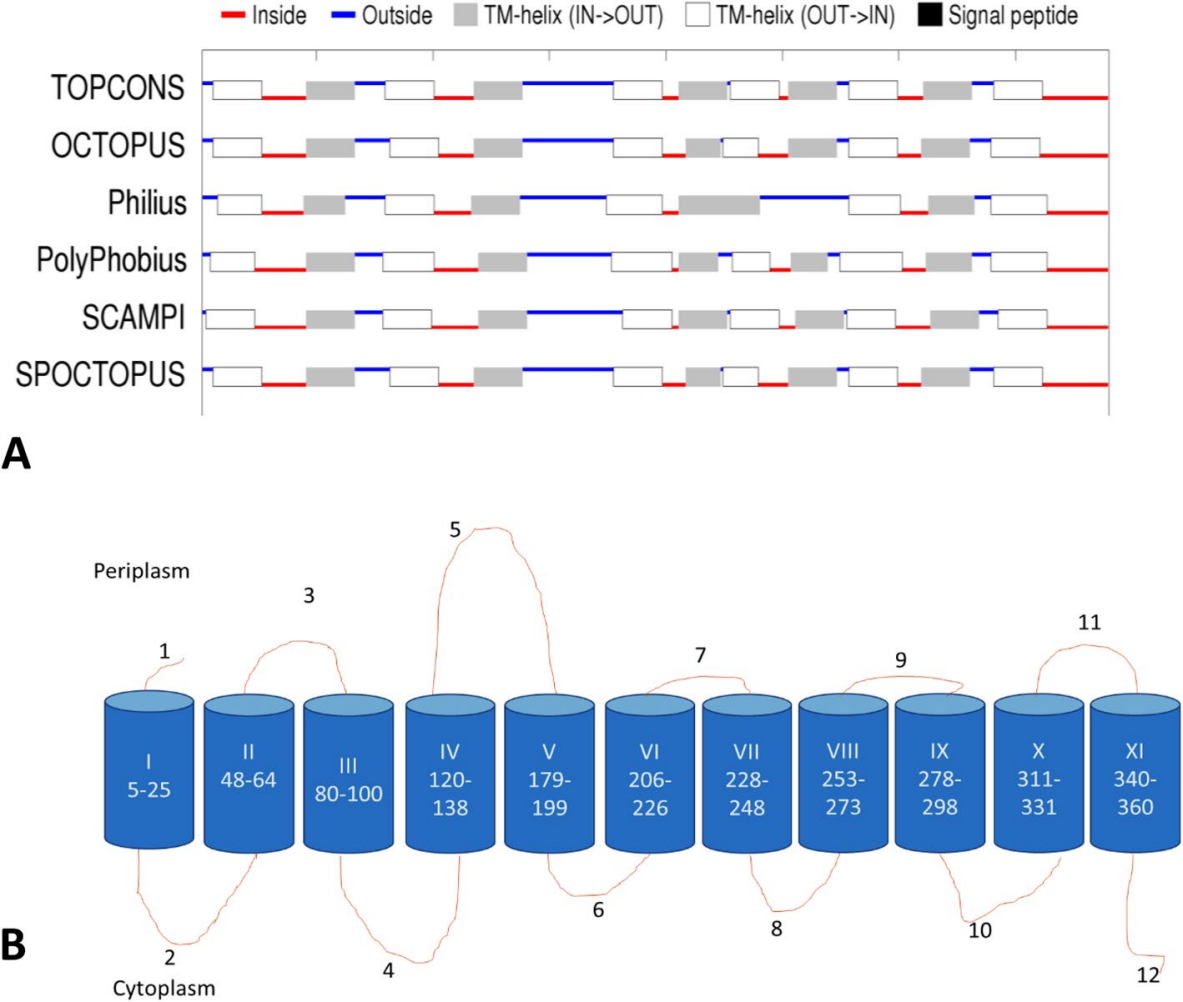
OacB topology model predicted by TOPCON. **A** Topology prediction of OacB. The red lines indicate ‘inside’, blue lines indicate ‘outside’; grey boxes indicate ‘Transmembrane(TM)-helix inside-out’ and empty boxes indicate ‘TM-helix outside-in’. **B** The consensus topology model of OacB is generated by the prediction programs. OacB is predicted to have 11 transmembrane (TM) domains, 12 loops with periplasmic N- and cytoplasmic C- termini. Roman numerals represent the TM domains, whereas Arabic numerals represent the loops in periplasm and cytoplasm

Five topology-predicting programs were used to build a working model of OacB, which use an algorithm based on Hidden Markov method. Philius predicted nine transmembrane helices, while all other four programs predicted OacB to have 11 transmembrane helices. This discrepancy could be because all other programs except Philius use protein sequences derived from multiple sequence alignment (MSA), whereas Philius uses only one protein sequence as input [11]. Based on this analysis, a consensus model of OacB having 11 transmembrane helices and 12 loops with the longest loop five at the periplasmic side was built. In addition, all five programs defined the orientations of N-terminus and C-terminus as periplasmic and cytoplasmic, respectively (Fig. 1).

### OacB similarities with other proteins

**Fig. 2:**
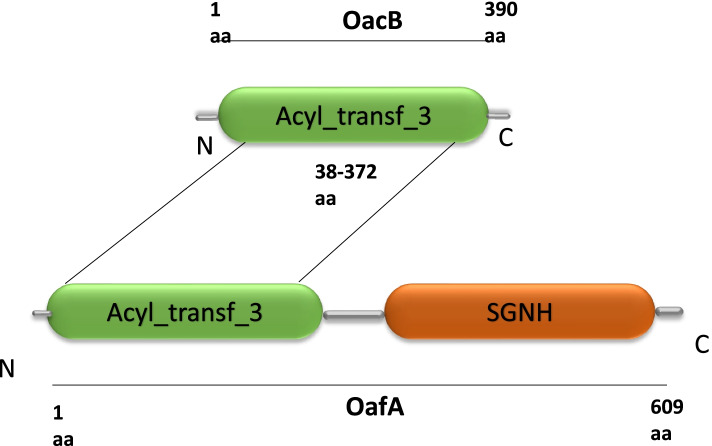
Domain architecture of OacB. Acyl_trans_3 domain of OacB spans from 38 to 372 amino acids. Blast hits identified OacB as similar to OafA (609 amino acids) containing acyltransferase and SGNH-hydrolase domains. [amino acid: aa]

The bioinformatics tools NCBI-CDD search revealed that OacB and its homologues are clustered under protein superfamily COG1835 or acyl_trans_3 (AT3), having a functional conserved acetyltransferase domain spanned from 38 to 372 amino acids (Fig. 2) [7]. The proteins having an acyl_tranf_3 domain are not uncommon in prokaryotes and eukaryotes and are involved in acylation modifications. The OacB protein BLAST showed similarities with a domain corresponding AT3 domain (Pfam 01757) of OafA. However, the SGNH hydrolase type esterase domain (Superfamily: SSF52266) of OafA was absent in OacB.

Proteins containing the SGNH domain along with AT3 or having either of the domains are involved in acetyltransferase activity [8, 13, 14]. Multiple alignments of OacB with acetyltransferases of other 16 evolutionarily related bacteria revealed that OacB shares identity with acetyltransferase proteins from *E. coli, Pseudomonas spp, Shigella spp*, *Acinetobacter baumanii*, *Flavobacterium,* and several other bacterial AT3 proteins (Additional Fig. 1). The pairwise alignment of OacB with a similar enzyme, O-acetyltransferase (Oac) has shown conservation of critical arginines R116 and R119 in cytoplasmic loop 4 which correspond to R73 and R76 in cytoplasmic loop 3 of Oac, respectively [3].

**Fig. 3:**
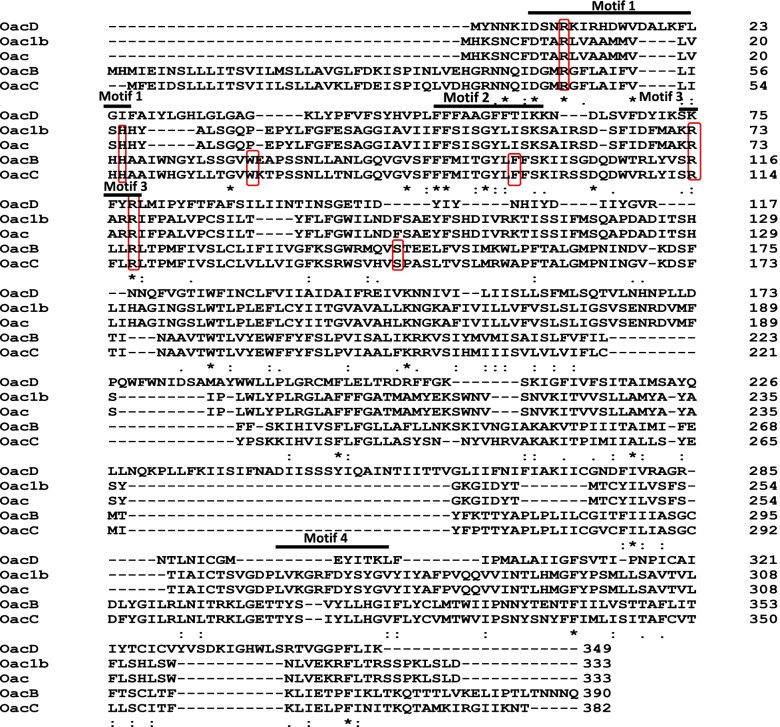
Multiple alignments of acetyltransferases (Oac, OacB, OacC, OacD, and Oac1b) from *Shigella flexneri* strains. The alignment was done using Clustal Omega. Asterisks and dots represent the amino acids that are identical or similar, respectively. Black lines above amino acids indicate three conserved motifs. Boxes with red outlines showing critical residues (R47, H58, W71, F98, R116, R119, and S146) identified in OacB

Furthermore, protein sequences of O-acetyltransferases of *S. flexneri* (Table 1) were obtained from GenBank to perform multiple alignments (Fig. 3). Three motifs DGxRGxLAxxVxxHH, FFxITG (YorF) LFxxK and SRxxR were found to be present in OacB, Oac1b, Oac, and OacC (Fig. 3). Moreover, two arginines found to be critical in Oac function (R73 and R76) were also conserved in all acetyltransferases of *S. flexneri* belonging to different serotypes. The conservation of the motifs and other amino acids in acetyltransferases of *S. flexneri* warrants their conserved role in *O*-acetylation. Additionally, OacC showed a marked 97% identity with OacB among all others whereas, OacD showed the least identity (13%) when compared in pairwise alignment using CLUSTAL Omega. Both OacB and OacC acetylate Rha III moiety of O-antigen in various serotypes of *S. flexneri* and homology between them indicates their close relationship. Oac and Oac1b showed 25% identity with OacB.

**Table 1:** *O*-acetyltransferases of *S. flexneri* from different serotypes

Gene.	Serotypes	Acetylated residue	Phage involved	Reference
*oac*	1b,3a,3b,4b and 7b	2-O-acetylation at Rhamanose(Rha) I	Sf6	[3]
*oacB*	1a, 1b, 2a, 5a, Y, 6 and 1c. 2a,3a, Y and Yv	¾ O-acetylation of RhaIII Position 6 of N acetyl glucosamine (GlcNAc)	Sf101	[7]
*oacC*	Serotype 6	¾ O-acetylation RhIII	Phage-like structure	[15]
*oacD*	Serotype 2	6-O-acetylation of N-acetylglucosamine (GlcNAc)	SfII	[16]
*oac1b*	1b	2-O acetylation at RhaIII	Novel phage	[17]

### Identification of critical residues in OacB

Multiple residues in OacB were mutated to alanine using SDM. The rationale for the selection of residues for mutagenesis largely was their conservation in acetyltransferases and chemical properties. However, several non-conserved residues were also selected to probe their role in OacB function. Details of residue location and reason for selection are summarized in Table 2. A total of 22 charged residues were mutated to alanine, which is a neutral amino acid. Alanine was chosen as a replacement of an amino acid in OacB, as it maintains the native spatial structure of the protein. pNV2132 harboring wild-type *oacB* and erythromycin resistance genes in pBAD *Myc*-HisA vector (Additional file 3) was used as a template to create single/double amino acids point mutations. For mutagenesis, aspartic acid 44 (D44) and basic amino acid arginine 47 (R47) were selected in cytoplasmic loop 2, which are part of the conserved motif 1. Aromatic residue tryptophan 71 (W71) from periplasmic loop 3; tyrosine 96 (Y96) and phenylalanine 98 (F98) polar residue from TM III were selected. Arginine 116 (R116) and 119 (R119) were selected in cytoplasmic loop 4. Moreover, residues of different charges were also mutated to alanine; proline 122 (P122) in TM IV; lysine 156 (K156) in long loop five; aromatic tryptophan 183 and threonine 184 (WT183–184); glutamic acid 188 (E188), and aromatic pair of tyrosine 191 and phenylalanine 192 (YF191–192) in TM V and histidine 320 (H320) in TM X. Details of the reason of residue selection and location are summarized in Table 2.

**Table 2:** Selection of different residues and results of slide agglutination assay using specific antisera to 3/4 *O*-acetylation against *S. flexneri* strains expressing different site-directed mutants of OacB

***S. flexneri***	Residue mutated to Alanine	Location in OacB	Reason for mutation	Agglutination level^a^	Functionality
SFL1691	N/A			–	
SFL1683	N/A			+++	Yes
SFL2591	D44	Cytoplasmic loop 2	Part of conserved Motif 1	+++	Yes
SFL2583	R47	Cytoplasmic loop 2	Part of conserved Motif 1	–	No
SFL2595	H58	TM II	Conserved in motif 1	–	No
SFL2584	W71	Periplasmic loop 3	Not conserved	–	No
SFL2596	V87	TM III	conserved	+++	Yes
SFL2588	Y96	TM III	Part of conserved motif 2	+++	Yes
SFL2589	F98	TM III	Part of conserved motif 2	–	No
SFL2585	R116	Cytoplasmic loop 4	Part of conserved motif 3	–	No
SFL2586	R119	Cytoplasmic loop 4	Part of conserved motif 3	–	No
SFL2593	P122	TM IV	Conserved	+++	Yes
SFL2620	S139	Periplasmic loop five	Not conserved Charge and part of large loop five	+	Yes
SFL2619	G140	Periplasmic loop five	Not conserved part of large loop five	+++	Yes
SFL2617	S146	Periplasmic loop 5	Not conserved part of large loop five	–	No
SFL2621	S153	Periplasmic loop five	Not conserved part of large loop five	+	Yes
SFL2587	K156	Periplasmic loop 5	Polar not conserved	+++	Yes
SFL2618	G164	Periplasmic loop five	Not conserved	++	Yes
SFL2616	D173	Periplasmic loop five	Not conserved	+++	Yes
SFL2622	S174	Periplasmic loop five	Not conserved part of large loop five	+++	Yes
SFL2592	E188	TM V	Not conserved	+++	Yes
SFL2594	FY191–192	TM V	Not conserved	+++	Yes
SFL2597	WT183–184	TM V	Not conserved	+++	Yes
SFL2590	H320	TM X	Not conserved catalytic residue	+++	Yes

^a^Agglutination levels were detected as being either strong (+ + +), medium (+ +), weak (+), or no agglutination (−). The absence of agglutination shows OacB is not functional or not present

Putative loop five in OacB is predicted to be the largest loop comprised of 40 amino acids. Most of the residues in loop five were not found to be conserved in acetyltransferases of other bacteria (Additional Fig. 1). However, the long loops have been found to play important roles in glucosyltransferases [18, 19]. Hence residues in loop five of OacB were targeted for mutagenesis to determine their role in OacB function [18, 20]. Overall, seven mutations were carried out in loop five. These included polar amino acid serine 139 (S139), glycine 140 (G140), serine 146 (S146), serine 153 (S153), glycine 164 (G164), an acidic aspartic acid 173 (D173), and serine 174 (S174).

### Functionality assay

**Fig. 4:**
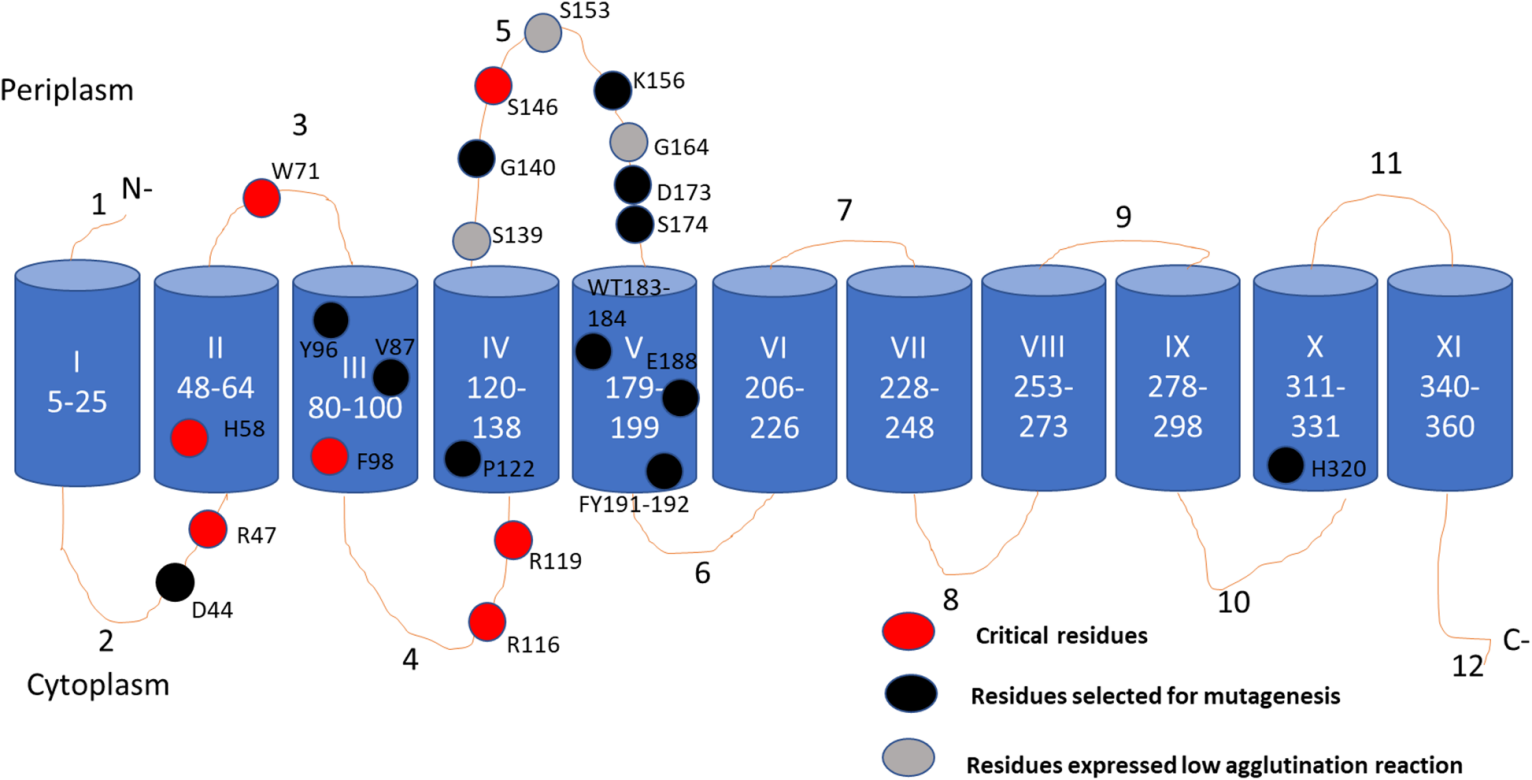
Topology model of OacB showing amino acids selected for site-directed mutagenesis. Topology model showing amino acids selected for mutagenesis. Red circles indicate amino acids critical for OacB whereas, black circles indicate non-critical residues. Grey coloured circle indicating residues showed slow agglutination reaction in slide agglutination test using serotype-specific antisera

To confirm the function of OacB in mutants, slide agglutination was performed using 3/4 O-acetyl-specific antiserum. Mutant plasmids were electroporated into serotype 1c strain SFL1691 for functional assessment by conversion of serotype 1c into 1c “variant” strain due to the catalytic action of OacB. *S. flexneri* 1c serotype “variant” strain SFL1683 harboring *oacB* wild type gene was used as a positive control. Seven strains namely SFL2583 (mutation R47), SFL2595 (mutation H58), SFL2584 (mutation W71), SFL2589 (mutation F98), SFL2585 (mutation R116), SFl2586 (mutation R119) and SFL2617 (mutation S146) failed to agglutinate with the antiserum. Whereas medium reaction (++) for SFL2618 (mutation G164) and low agglutination reactions (+) were observed in strains SFL2620 (mutation S139), and SFL2621(mutation S153) (Table 2 and Additional Fig. 2). Locations of critical residues are highlighted in the 2D model of OacB (Fig. 4)**.** However, other mutants D44, V87, Y96, P122, K156, D173, S174, WT183–184, E188, and FY191–192 reacted positively with the serotype-specific antiserum indicating having a functional enzyme.

### Role of residues in the assembly of protein in the membrane

**Fig. 5:**
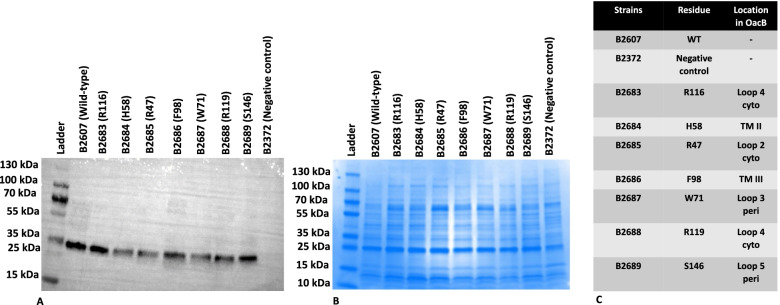
SDS-PAGE and western blot of membrane protein of OacB. Membrane proteins of mutants of OacB were prepared and subjected to western blot using anti-His antibodies. An equal amount of ~ 25 μg of each protein was loaded onto an SDS-PAGE gel. B2607 (wild type = WT) was used as positive control and B2372 was used as a negative control. Pre-stained PageRuler™ was used as a molecular mass marker. OacB protein band is indicated running at 35 kDa. (Membrane proteins tend to run differently onto SDS-PAGE from their actual molecular weight due to ‘gel shift’). **B**, Equal amounts of proteins loaded onto another SDS-PAGE gel and Coomassie-stained. **C**, Table summarizing location of critical residues in OacB. (Figure shows cropped images of the western blot and the Coomassie-stained gel. The uncropped full-length images of these are shown in Additional Fig. 3)

To confirm the assembly and synthesis of non-functional mutants of OacB in the plasma membrane, membrane proteins of all the mutant strains were prepared. An equal amount (~ 25 μg) protein of all OacB non-functional mutants and wild-type OacB proteins was subjected to SDS-PAGE gel. Western blot using anti-His antibodies was performed. All the non-functional mutants of OacB were found to be assembled in the membrane as the wild-type protein (Fig. 5 and Additional Fig. 3).

## Discussion

In the present study, several critical residues in OacB were identified using site-directed mutagenesis (SDM) and agglutination assay. Alignment of proteins having homology with OacB helped identify several conserved residues present in TM helical regions and periplasmic and cytoplasmic loops. Selected residues were then subjected to SDM to confirm their role in OacB function. The functionality assays revealed the presence of seven critical residues in OacB. The assembly and synthesis of all the non-functional mutants of OacB in the plasma membrane were confirmed using western blotting.

The non-essential residues identified in OacB were D44, Y96, V87, K156, P122, D173, S174 WT183–184, E188, and FY191–192. The critical amino acids detected through negative agglutination reaction include three arginine residues (R47, R116, and R119); phenylalanine (F98); tryptophan residue (W71); histidine (H58), and serine (S146). The importance of arginine residues in protein assembly and catalysis is well-documented [21, 22]. Hence, we targeted three conserved arginine residues for substitution with alanine R47, R116, and R119 to confirm their role in OacB. The results revealed that the replacement of any of the three arginine residues affected the function of the protein, as the mutants failed to react with serotype-specific antiserum. Residues R47, R116, and R119 are also found to be fairly conserved among proteins containing single (AT3) /double (AT3-SGNH) domain (s) acetyltransferases in other domains of life [8, 13]. Another study investigating topological features of Oac revealed that arginines can play diverse roles depending on their location in the protein, and identified residues important for Oac function present in the conserved cytoplasmic RxRR motif [3, 21]; R75 and R76 were found to play a structural role, whereas R73 was attributed to the catalytic role. In this study, two arginine residues of OacB, R116, and R119 in cytoplasmic loop 4, corresponding to Oac residues R73, and R76, respectively, were mutated to alanine. The functionality assays revealed a non-functional protein, indicating roles of these residues in function of OacB. Recently, corresponding /equivalent arginine residues R69 and R72 in transmembrane helix I are also found to play an essential role in dual-domain protein OafA, which acetylates abequose residue of the O-antigen in *Salmonella enterica serovar Paratyphi* [8]*.* Similarly, in another protein, OafB, when corresponding arginine R71 and R74 in conserved RxxR motif were replaced with alanine, the resultant protein failed to complement the short O-antigen phenotype [13]. Moreover, the substitution of R47 in OacB also affected the functionality and hence suggests its importance in OacB. However, the role of the corresponding residue to R47 was not evaluated in Oac. R47, R116, and R119 are conserved in almost 30 other acetyltransferases belonging to other bacteria and plants acetyltransferases [8]. Among five acetyltransferases of *S. flexneri*, both arginines (R116 and R119) are found to be conserved in all with only one difference that instead of arginine (R) another similar amino acid lysine (K) in OacD is present at the corresponding position of R119 of OacB.

Arginines have been reported as catalytic residue and serve as a general base catalyst [23]. In a study, the role of the arginine residue (R359) is explained as the oxyanion hole forming residue in the PatB1 enzyme, which belongs to SGNH domain-containing protein and responsible for O-acetylation of secondary cell wall polysaccharides in *Bacillus cereus* [22]. When R359 was mutated to alanine to investigate its role in an experiment, the variant protein failed to perform esterase or transferase activities, indicating importance of R359 as an active site residue. It appears that cytoplasmic R116 in OacB might be involved in a catalytic role, whereas R47 and R119 might have structural roles. However, further biochemical experiments are required to confirm the roles played by these two arginines in OacB.

In this study, W71 present in the periplasmic loop 3, was targeted for mutagenesis because of its conserved position in other acetyltransferases. The results obtained in this study showed the critical role of W71 in the function of OacB. Hence, it is yet to be determined that W71 plays either a catalytic or structural role in OacB. Tryptophan is an aromatic amino acid with large cyclic side chains, and capable of making protein to protein/ligand interactions with their neighbouring residues by its large hydrophobic polar side chain [24, 25]. Replacement of W71 residue in OacB resulted in the loss of protein function and maybe this residue was critical to maintain interactions with nearby residues and its mutation resulted in structural changes in OacB.

Another important residue identified in the current study was F98. The phenylalanine side chain is considered critical for full catalytic activity in a protein. A conserved phenylalanine residue F98 in transmembrane III was replaced with alanine in OacB. Mutation of this residue affected OacB function which was discernible with the slide agglutination test. In a study to evaluate the role of phenylalanine residue, Shmara et al.*,* performed alanine scanning mutagenesis to the aminoglycoside 6′ -N-acetyltransferase type 1b that confers resistance to aminoglycoside antibiotics, and the replacement of F171 resulted in the loss of resistance to kanamycin and amikacin, in the resultant derivatives [26]; they postulated the participation of this residue in acceptor substrate specificity. Phenylalanine is hypothesized to play a central role in the alignment of the acetyl group for the transfer to the substrate [27]. Furthermore, the study on arylamine acetyltransferases NAT1 and NAT2 supported that F125 residue involved in forming active site and played role in substrate selectivity in human arylamine acetyltransferases NAT1 and NAT2 [28]. A similar role of F98 can be attributed in OacB due to its location close to conserved arginine residues (R116 and R119).

The role of four serine residues S139, S146, S153, and S174 found in large periplasmic loop 5 in OacB was also investigated. Serine residues are found to be critical for activity in many proteins [3, 8, 29]. Conserved serine residues in Ser-Lys dyad or catalytic triad in SGNH hydrolases are involved in catalytic activities in enzymes. In a study on the O-acetylation of secondary cell wall polysaccharide (SCWP) by PatB1, serine 337 was identified as a catalytic nucleophile in the crystal form of PatB1. Similarly, in another study serine residue in OafA of *Salmonella enterica serovar Typhi* (O:5) was found to constitute an oxyanion hole in the C-terminal SGNH periplasmic domain [30]. In our study, when four non-conserved serine residues S139, S146, S153, and S174 present in the large periplasmic loop 5 were replaced with an alanine, and functionality of each mutant was tested in slide agglutination assay, only S146 mutant (SFL2617) failed to agglutinate with the serotype-specific antiserum. Amino acid, S174 was replaced with alanine without affecting the function of OacB, whereas mutants S139 and S153 showed low agglutination reactions. The substitution of the latter two may have resulted in disruption of the catalytic site involved with the substrate interaction in the periplasmic loop 5. The complete absence of function observed in the case of S146 mutation indicates the essential role played by this residue. Considering the O-acetylation occurs in periplasm we hypothesize that residue S146 in the large periplasmic loop 5 might be responsible for adding an acetyl group to the Rha III moiety of O-antigen. The critical role of large periplasmic loops has previously been reported in glucosyltransferases of *S. flexneri* [19, 31].

Furthermore, in our study when two glycines in large periplasmic loop 5 were mutated to alanine, medium agglutination reaction was observed for G164 as compared to the positive control, whereas G140 agglutinated strongly. Glycine is a non-polar amino acid having a single hydrogen atom as its side chain instead of carbon. This feature permits glycine to fit into tight turns of the protein that might limit other amino acids. The exact role of glycine in OacB is not yet clear and it is thought that the reduced agglutination reaction detected for mutant G164 might be due to the structural connection of this residue with an active site residue (S146) and when mutated to alanine the interaction with active site residues was affected.

The definite role of Histidine 58 (H58) in OacB is not clear, however, the conserved status of this residue in thirty other acetyltransferases found in other organisms indicates its essential role [8]. In our study, the loss of function of OacB after replacing H58 with an alanine confirmed its critical role. In OafA and OatA, histidine is part of the catalytic triad of the SGNH domain and is involved in catalysis [8, 32]. Moreover, an equivalent residue H25 in OafA of *Salmonella enterica serovar Typhimurium* has recently been found to play a role in processing the bound acetate to arginine residue-R14 in TMH1 and transferring it to the periplasmic side of the inner membrane [8].

## Conclusion

This study assessed the role of conserved and non-conserved amino acids in OacB and has yielded valuable information on the functional roles played by individual amino acids. Functionally important residues were identified in transmembrane domains, cytoplasmic and periplasmic loops in OacB. Substitution of R47, R116, R119, H58, W71, F98, and S146 resulted in loss of protein function. Our results reiterate the importance of strictly conserved arginine residues in acetyltransferases. The knowledge gained on the critical residues and domains of this protein can help support future studies to understand the O-antigen modification mechanism by OacB and subsequently other acetyltransferases.

## Supplementary Information

**Additional file 1.**

**Additional file 2.**

**Additional file 3.**

**Additional file 4.**

**Additional file 5.**

**Additional file 6.**

**Additional file 7.**

**Additional file 8.**

## Data Availability

The datasets analysed during the current study are available in the GenBank repository and accession numbers to datasets are as follows: OacB protein from Sf101 phage (AII27840.1); Oac (P23214); OacC (EFW62204); OacD (WP_000282635); Oac1b (AFM56533.1); *Escherichia coli (*EFC1419592.1); *Rhodobacter capsulatus* (WP_023921362.1); *Competibacter denitrificans* Run_A_D11 (WP_071243973.1); *Methyloversatilis thermotolerans* (WP_018410755.1); *Paraburkholderia heleia* (WP_052405754.1); *Pseudomonas_corrugata* (WP_055136861.1); *Pseudomonas_aeruginosa* (DK1WP_071537607.1); *Pseudomonas fluorescens* F113 (WP_014337242.1); *Flavobacterium columnare* ATCC_49512 (WP_014166569.1); *Dechloromonas aromatica* RCB (AAZ46617.1); *Flavobacterium columnare* (WP 060383091.1); *Cupriavidus metallidurans* CH34 (WP_011517319.1); *Burkholderiales bacterium* GJ-E10 (BAP89224.1); *Acinetobacter baumannii* 299505 (WP32009270.1); *Azospirillum lipoferum* (WP_012978429.1).

## Abbreviations

OacB/oacB: O-acetyltransferase B
Gtr/gtr: Glucosyltransferases
LPS: Lipopolysaccharide
GlcNAc: N-acetylglucosamine
AT3: Acetyltransferase 3
CIAP: Calf intestinal alkaline phosphatase
SDM: Site-directed mutagenesis
Rha: Rhamnose
PCR: Polymerase chain reaction
SDS: Sodium-dodecyl sulphate
PVDF: Polyvinylidene fluoride
PBS: Phosphate buffer saline
aa: Amino acid
