# Supplementary material for: Identification of critical residues of O-antigen-modifying O-acetyltransferase B (OacB) of Shigella flexneri
Source: BMC Mol Cell Biol. 2022 Mar 24;23:16. doi: 10.1186/s12860-022-00415-8 (PMC8952252; doi:10.1186/s12860-022-00415-8)
Supplement: Supplementary file 1 — Additional file 1. [file 12860_2022_415_MOESM1_ESM.doc]

**Table S1: *E. coli* strains used/created in this study**

| **Strain** | **Genotype** | **Source / Reference** |
| --- | --- | --- |
| JM109 | *rec*A1, *sup*E44, *end*A1, *hsd*R17, *gyr*A96, *rel*A1 *thi* ∆(lac-*pro*AB) [F’ *tra*D36, *pro*AB+ *lac*Iq, *lacZ*∆M15 | Yanisch *et al*., 1985 |
| XL1-Blue MRF | *sup*E44, *hsd*R17, *recA*1, *end*A1, *gyrA*96, *thi*-1 *relA*1, *lac* [F’ *pro*AB, *lacIq*Z∆M15 Tn*10* (Tetr)] | Stratagene |
| TOP10 | F–*mcr*A Δ, *(mrr-hsd* RMS-*mcr*BC), φ80*lac*ZΔM15 Δ*lac*X74, *rec*A1, araD139, Δ(*ara*A-l*eu*)7697 *gal*U *gal*K, *rps*L, *end*A1, *nup*G | Invitrogen |
| B2575 | JM109 carrying pNV2111 | This study |
| B2596 | JM109 carrying pNV2132 | This study |
| B2607 | TOP10 carrying pNV2132 | This study |
| B2619 | XLI carrying pNV2146 | This study |
| B2620 | XLI carrying pNV2147 | This study |
| B2621 | XLI carrying pNV2148 | This study |
| B2622 | XLI carrying pNV2149 | This study |
| B2623 | XLI carrying pNV2150 | This study |
| B2624 | XLI carrying pNV2151 | This study |
| B2625 | XLI carrying pNV2152 | This study |
| B2626 | XLI carrying pNV2153 | This study |
| B2627 | XLI carrying pNV2154 | This study |
| B2628 | XLI carrying pNV2155 | This study |
| B2629 | XLI carrying pNV2156 | This study |
| B2630 | XLI carrying pNV2157 | This study |
| B2631 | XLI carrying pNV2180 | This study |
| B2632 | XLI carrying pNV2178 | This study |
| B2633 | XLI carrying pNV2179 | This study |
| B2683 | TOP10 carrying pNV2147 | This study |
| B2684 | TOP10 carrying pNV2178 | This study |
| B2685 | TOP10 carrying pNV2146 | This study |
| B2686 | TOP10 carrying pNV2152 | This study |
| B2687 | TOP10 carrying pNV2148 | This study |
| B2688 | TOP10 carrying pNV2149 | This study |
| B2689 | TOP10 carrying pNV2171 | This study |
