# Supplementary material for: Identification of critical residues of O-antigen-modifying O-acetyltransferase B (OacB) of Shigella flexneri
Source: BMC Mol Cell Biol. 2022 Mar 24;23:16. doi: 10.1186/s12860-022-00415-8 (PMC8952252; doi:10.1186/s12860-022-00415-8)
Supplement: Supplementary file 2 — Additional file 2. [file 12860_2022_415_MOESM2_ESM.docx]

**Table S2: *S. flexneri* strains used/created in this study**

| **Strain** | **Description** | **Source** |
| --- | --- | --- |
| SFL1683 | *Shigella flexneri* 1c wild type lysogenic strain (*oacB* positive) | El-Gendy et al.1999b |
| SFL1691 | *Shigella flexneri* 1c wild type strain negative for *oacB* | El-Gendy et al.1999b |
| SFL2572 | SFL1691 carrying pNV2132 | This study |
| SFL2583 | SFL1691 carrying pNV2146 | This study |
| SFL2584 | SFL1691 carrying pNV2148 | This study |
| SFL2585 | SFL1691 carrying pNV2147 | This study |
| SFL2586 | SFL1691 carrying pNV2149 | This study |
| SFL2587 | SFL1691 carrying pNV2150 | This study |
| SFL2588 | SFL1691 carrying pNV2151 | This study |
| SFL2589 | SFL1691 carrying pNV2152 | This study |
| SFL2590 | SFL1691 carrying pNV2154 | This study |
| SFL2591 | SFL1691 carrying pNV2153 | This study |
| SFL2592 | SFL1691 carrying pNV2155 | This study |
| SFL2593 | SFL1691 carrying pNV2156 | This study |
| SFL2594 | SFL1691 carrying pNV2157 | This study |
| SFL2595 | SFL1691 carrying pNV2178 | This study |
| SFL2596 | SFL1691 carrying pNV2179 | This study |
| SFL2597 | SFL1691 carrying pNV2180 | This study |
| SFL2616 | SFL1691 carrying pNV2172 | This study |
| SFL2617 | SFL1691 carrying pNV2171 | This study |
| SFL2618 | SFL1691 carrying pNV2173 | This study |
| SFL2619 | SFL1691 carrying pNV2174 | This study |
| SFL2620 | SFL1691 carrying pNV2175 | This study |
| SFL2621 | SFL1691 carrying pNV2176 | This study |
| SFL2622 | SFL1691 carrying pNV2177 | This study |
