# Supplementary material for: Identification of critical residues of O-antigen-modifying O-acetyltransferase B (OacB) of Shigella flexneri
Source: BMC Mol Cell Biol. 2022 Mar 24;23:16. doi: 10.1186/s12860-022-00415-8 (PMC8952252; doi:10.1186/s12860-022-00415-8)
Supplement: Supplementary file 3 — Additional file 3. [file 12860_2022_415_MOESM3_ESM.docx]

**Table S3: Plasmids used/created in this study**

| **Plasmid** | **Characteristics** | **Source/Reference** |
| --- | --- | --- |
| pBAD/*Myc*-His A | pBAD/*Myc*- His plasmid derived from PBR322 | Invitrogen |
| pBAD/*Myc*-His/lacZ | pBAD/*Myc*-His*/lac*Z is a 7242 bp control vector containing the gene for B-galactosidase fused to the C-terminal peptide | Invitrogen |
| pNV2111 | Wild type *oacB cloned into* pBAD/*Myc*-HisA vector using *Xho*I and *EcoR*I sites | This study |
| pNV2132 | Erythromycin resistance gene introduced at *Sph*I site in pNV2111 | This study |
| pNV2146 | pNV2132 with OacB Arginine47 mutated to Ala | This study |
| pNV2147 | pNV2132 with OacB Arginine116 mutated to Ala | This study |
| pNV2148 | pNV2132 with OacB Tryptophan71 mutated to Ala | This study |
| pNV2149 | pNV2132 with OacB Arginine119 mutated to Ala | This study |
| pNV2150 | pNV2132 with OacB Lysine156 mutated to Ala | This study |
| pNV2151 | pNV2132 with OacB Tyrosine96 mutated to Ala | This study |
| pNV2152 | pNV2132 with OacB Phenylalanine98 mutated to Ala | This study |
| pNV2153 | pNV2132 with OacB Aspartic acid44 mutated to Ala | This study |
| pNV2154 | pNV2132 with OacB Histidine320 mutated to Ala | This study |
| pNV2155 | pNV2132 with OacB Glutamic acid188 mutated to Ala | This study |
| pNV2156 | pNV2132 with OacB Proline122 mutated to Ala | This study |
| pNV2157 | pNV2132 with OacB Phenylalanine-Tyrosine191-192 mutated to Ala/Ala | This study |
| pNV2178 | pNV2132 with OacB Histidine58 mutated to Ala | This study |
| pNV2179 | pNV2132 with OacB Valine87 mutated to Ala | This study |
| pNV2180 | pNV2132 with OacB Tryotophan-Threonine183-184 mutated to Ala/Ala | This study |
| pNV2171 | pNV2132 with OacB Seiner146 mutated to Ala | This study |
| pNV2172 | pNV2132 with OacB Aspartic acid173 mutated to Ala | This study |
| pNV2173 | pNV2132 with OacB Glycine164 mutated to Ala | This study |
| pNV2174 | pNV2132 with OacB Glycine140 mutated to Ala | This study |
| pNV2175 | pNV2132 with OacB Serine139 mutated to Ala | This study |
| pNV2176 | pNV2132 with OacB Serine153 mutated to Ala | This study |
| pNV2177 | pNV2132 with OacB Serine174 mutated to Ala | This study |
