# Supplementary material for: Identification of critical residues of O-antigen-modifying O-acetyltransferase B (OacB) of Shigella flexneri
Source: BMC Mol Cell Biol. 2022 Mar 24;23:16. doi: 10.1186/s12860-022-00415-8 (PMC8952252; doi:10.1186/s12860-022-00415-8)
Supplement: Supplementary file 4 — Additional file 4. [file 12860_2022_415_MOESM4_ESM.docx]

**Table S4: Cloning primers used in this study**

| **Primer Name** | **Primer Sequence *** | **Restriction site(s)** |
| --- | --- | --- |
| *oacB* pBADF | CCG**CTCGAG**GATGCA TAT GAT | *XhoI at 5’* |
| oacB pBAD R | CCT**GAATTC**CGTTGATTGTTGTT | *EcoRI at 3’* |
| EmF-SphI-Fwd | AAT**GCATGC**TAAGACGGTTCGTGTTCGT | *SphI* |
| EmR-SphI-Rev | AAT**GCATGC**CATAGAATTATTTCCTCCCG | *SphI* |

*Restriction sites are indicated in bold letters
