# Supplementary material for: Identification of critical residues of O-antigen-modifying O-acetyltransferase B (OacB) of Shigella flexneri
Source: BMC Mol Cell Biol. 2022 Mar 24;23:16. doi: 10.1186/s12860-022-00415-8 (PMC8952252; doi:10.1186/s12860-022-00415-8)
Supplement: Supplementary file 5 — Additional file 5. [file 12860_2022_415_MOESM5_ESM.docx]

**Table S5: Site-directed mutagenesis primers**

| **Primer name** | **Primer sequence^*^** |
| --- | --- |
| D44F | GAATAATCAAATAG**C**TGGAATGCGGGGGTTCTTAG |
| D44R | CTAAGAACCCCCGCATTCCA**G**CTATTTGATTATTC |
| R47F | CAAATAGATGGAATGG**CG**GGGTTCTTAGCAATTTTC |
| R47R | GAAAATTGCTAAGAACCC**CG**CCATTCCATCTATTTG |
| H58F | CTTATTCAT**GC**CGCAGCAATTTGG |
| H58R | CCAAATTGCTGCG**GC**ATGAATAAG |
| W71F | CTTGTCATCTGGAGTAGCGGAAGCACCTTCATCAAATC |
| W71R | GATTTGATGAAGGTGCTTCCGCTACTCCAGATGACAAG |
| V87F | GCCAAGTTGGTG**CT**TCATTCTTTTTTATG |
| V87R | CATAAAAAAGAATGA**AG**CACCAACTTGGC |
| Y96F | CTTTTTTATGATTACTGGT**GC**TCTGTTCTTTTCAAAG |
| Y96R | CTTTGAAAAGAACAGA**GC**ACCAGTAATCATAAAAAAG |
| F98F | GATTACTGGTTATCTG**GC**CTTTTCAAAGATTATCTC |
| F98R | GAGATAATCTTTGAAAAG**GC**CAGATAACCAGTAATC |
| R116F | GACAAGGCTTTATGTATCA**GC**ATTACTACGATTAACCC |
| R116R | GGGTTAATCGTAGTAAT**GC**TGATACATAAAGCCTTGTC |
| R119F | GTATCAAGATTACTA**GC**ATTAACCCCAATGTTC |
| R119R | GAACATTGGGGTTAAT**GC**TAGTAATCTTGATAC |
| P122F | GATTACTACGATTAACC**GC**AATGTTCATAGTTAGCCT |
| P122R | AGGCTAACTATGAACATT**GC**GGTTAATCGTAGTAATC |
| S139F | CATTGTAGGTTTTAAGGCT**GG**ATGGAGAATGCAGG |
| S139R | CCTGCATTCTCCATCCAG**CC**TTAAAACCTACAATG |
| G140F | CATTGTAGGTTTTAA**G**TCTGCATGGAGAATGC |
| G140R | GCATTCTCCAT**G**CAGACTTAAAACCTACAATG |
| S146F | GAATGCAGGTA**G**CCACAGAAGAGCTTTTTGTG |
| S146R | CACAAAAAGCTCTTCTGTGG**C**TACCTGCATTC |
| S153F | CAGAAGAGCTTTTTGTG**G**CAATAATGAAGTGG |
| S153R | CCACTTCATTATTG**C**CACAAAAAGCTCTTCTG |
| G164F | GCCATTCACTGCACTAG**C**TATGCCGAACATTAATGACG |
| G164R | CGTCATTAATGTTCGGCATAGCTA**G**TGCAGTGAATGGC |
| D173F | CGAACATTAATGACGTAAAAG**C**TTCATTTACTATCAATGC |
| D173R | GCATTGATAGTAAATGAA**G**CTTTTACGTCATTAATGTTCG |
| S174F | CATTAATGACGTAAAAGAT**G**CATTTACTATCAATGCCGCTGTAAC |
| S174R | GTTACAGCGGCATTGATAGTAAATG**C**ATCTTTTACGTCATTAATG |
| WT183-184 F | CTGTAACA**GC**G**G**CACTTGTATATGAATG |
| WT183-184 R | CATTCATATACAAGT**GC**C**G**CTGTTACAG |
| E188F | GTAACATGGACACTTGTATATG**C**ATGGTTCTTTTATTTTTC |
| E188R | GAAAAATAAAAGAACCATG**C**ATATACAAGTGTCCATGTTAC |
| FY191-192F | GTATATGAATGGTTC**GCTGCT**TTTTCTCTTCCGGTAATTTC |
| FY191-192R | GAAATTACCGGAAGAGAAAA**AGCAGC**GAACCATTCATATAC |
| H320F | GCGTTTACCTTCTG**GC**TGGGATATTCCTTTATTGC |
| H320R | GCAATAAAGGAATATCCCA**GC**CAGAAGGTAAACGC |

***^The substituted nucleotides are indicated in bold^**
