# Supplementary material for: Identification of critical residues of O-antigen-modifying O-acetyltransferase B (OacB) of Shigella flexneri
Source: BMC Mol Cell Biol. 2022 Mar 24;23:16. doi: 10.1186/s12860-022-00415-8 (PMC8952252; doi:10.1186/s12860-022-00415-8)
Supplement: Supplementary file 6 — Additional file 6. [file 12860_2022_415_MOESM6_ESM.pdf]

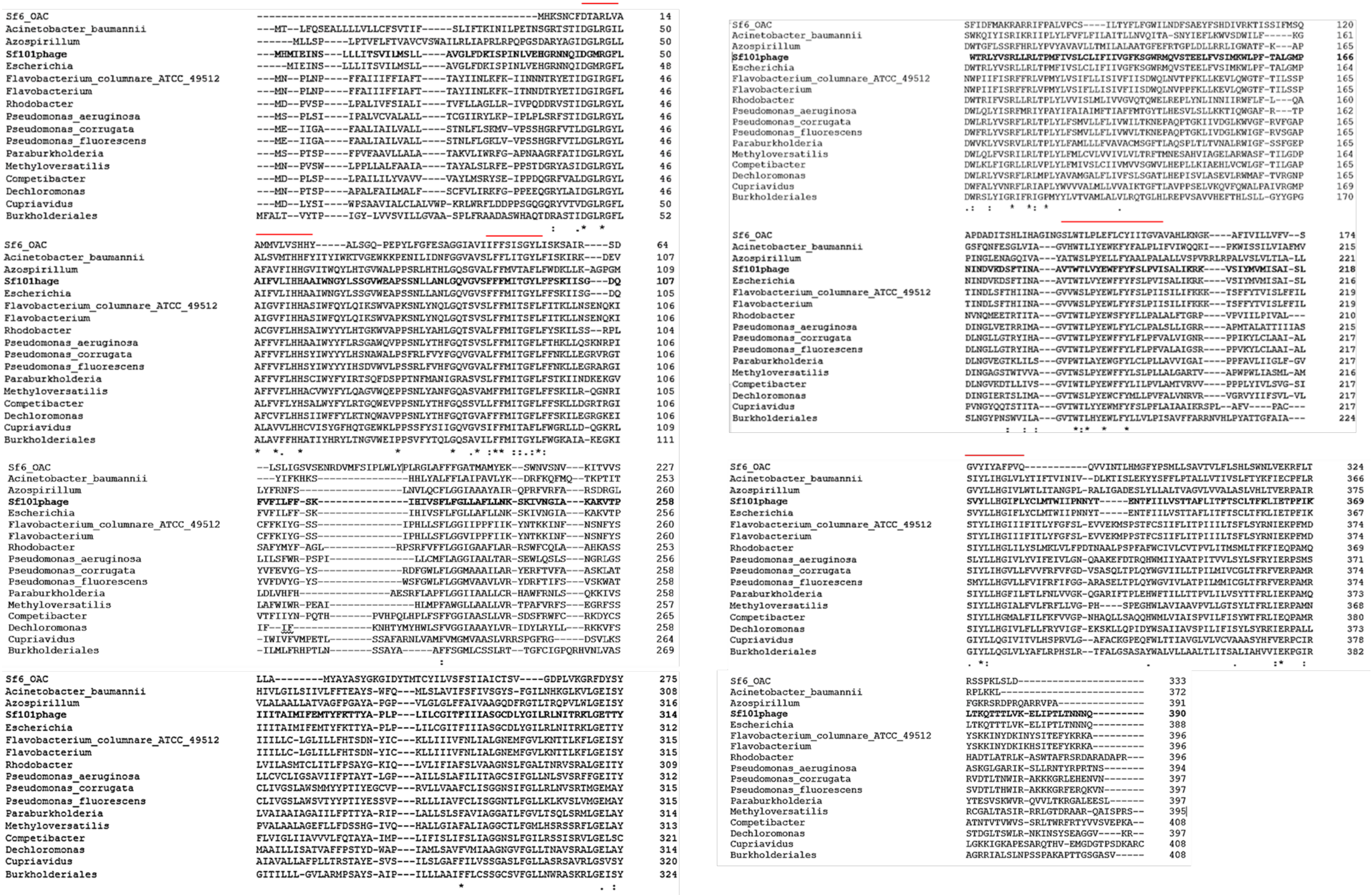

Sf6_OAC
Acinetobacter_baumannii
Azospirillum
Sf101phage
Escherichia
Flavobacterium_columnare_ATCC_49512
Flavobacterium
Rhodobacter
Pseudomonas_aeruginosa
Pseudomonas_corrugata
Pseudomonas_fluorescens
Paraburkholderia
Methyloversatilis
Competibacter
Dechloromonas
Cupriavidus
Burkholderiales

Additional Figure 1. Multiple alignments of Sf101 OacB with other acyltransferases. Clustal Omega was used to align OacB protein from Sf101 phage with its homologues in other species. Conserved motifs are shown by red lines on top. Amino acid sequence of OacB from Sf101 is shown in bold letters.
