# Supplementary material for: Identification of critical residues of O-antigen-modifying O-acetyltransferase B (OacB) of Shigella flexneri
Source: BMC Mol Cell Biol. 2022 Mar 24;23:16. doi: 10.1186/s12860-022-00415-8 (PMC8952252; doi:10.1186/s12860-022-00415-8)
Supplement: Supplementary file 7 — Additional file 7. [file 12860_2022_415_MOESM7_ESM.pdf]

Figure S2

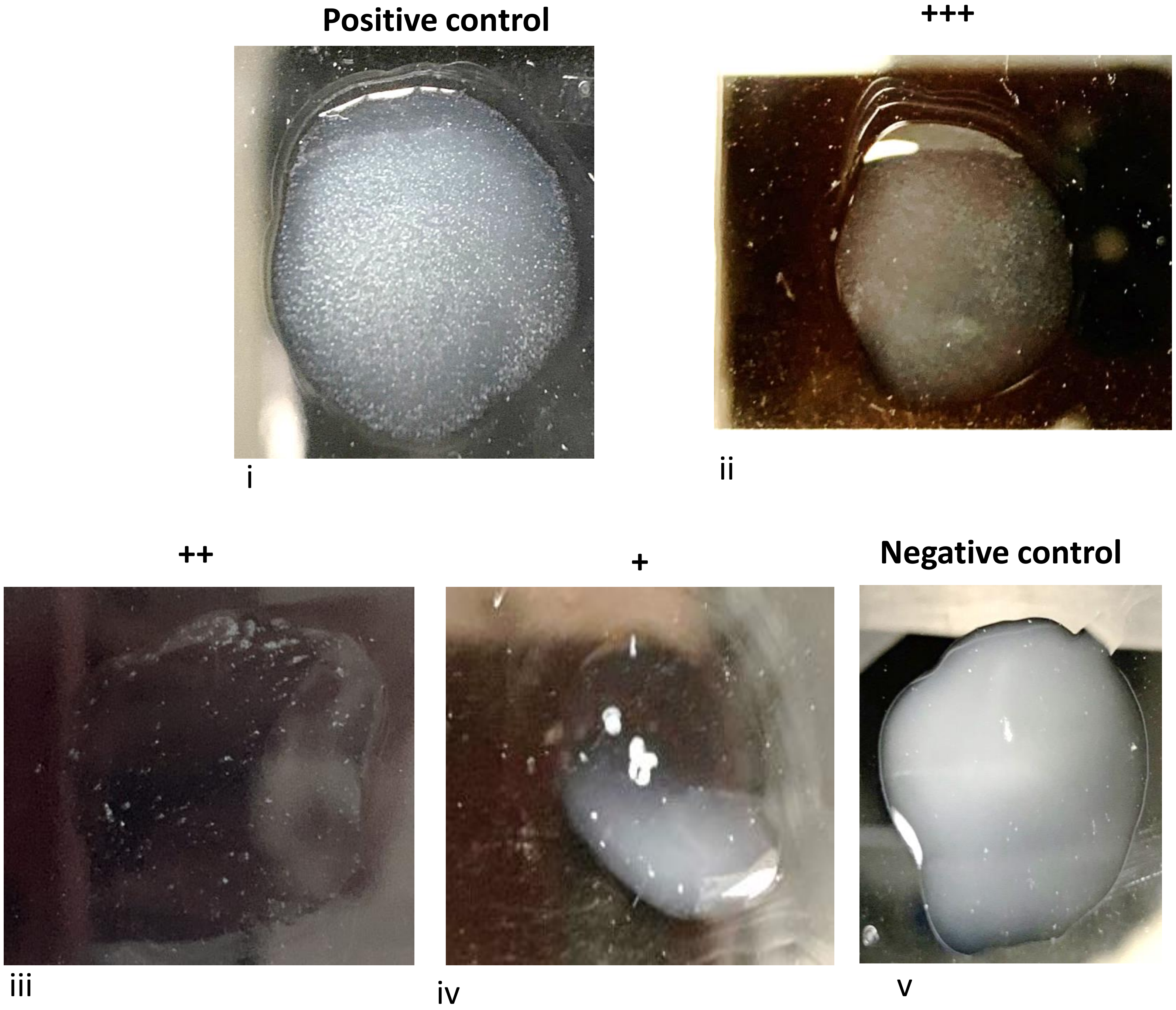

Functionality of OacB. A representative image is shown for each level of agglutination achieved with different OacB mutants in agglutination assays. To test the functionality of OacB, the mutagenized plasmids were electroporated into 1c strain SFL1691 (which was negative for *oacB* gene as confirmed by its genome sequence) to produce recombinant *S. flexneri* strains. The agglutination assays were performed by mixing a single isolated colony of the test strain with ¾ *O*-acetyl-specific antiserum on a glass slide. The slide was rotated back and forth for 60 seconds. Visible clumping of bacterial cells with the antiserum achieved within one minute of swirling was considered as positive, which in turn represented the presence of functional OacB. Levels of agglutination were compared against the clumping of positive control strain, SFL1683 (serotype 1c strain carrying *oacB* gene) within one minute of the addition of antiserum. Agglutination results recorded (panels ii-v) as high (+++), medium (++), low (+) or no agglutination (-). SFL1691 was used as a negative control (panel v) which showed no agglutination even after one minute of the addition of antiserum. The agglutination assays were performed in triplicates.
