# Supplementary material for: Identification of critical residues of O-antigen-modifying O-acetyltransferase B (OacB) of Shigella flexneri
Source: BMC Mol Cell Biol. 2022 Mar 24;23:16. doi: 10.1186/s12860-022-00415-8 (PMC8952252; doi:10.1186/s12860-022-00415-8)
Supplement: Supplementary file 8 — Additional file 8. [file 12860_2022_415_MOESM8_ESM.pdf]

Figure S3

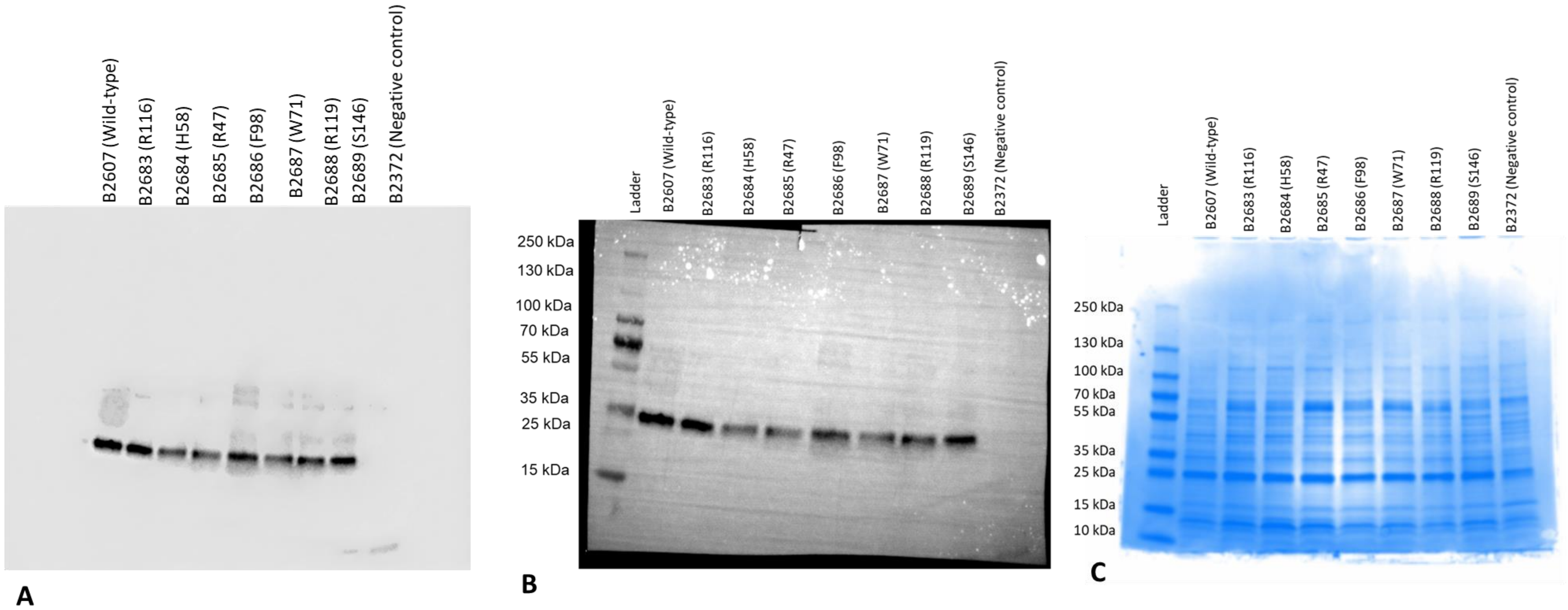

Full length western blot (A/B) with and without ladder; and Coomassie stained gel (C), corresponding to Figure 5 in the manuscript.
